# Genome Evolution from Random Ligation of RNAs of Autocatalytic Sets

**DOI:** 10.3390/ijms222413526

**Published:** 2021-12-16

**Authors:** Felix Broecker

**Affiliations:** Idorsia Pharmaceuticals Ltd., Hegenheimermattweg 91, CH-4123 Allschwil, Switzerland; felixbroecker@gmx.net

**Keywords:** genome evolution, ribozymes, RNA ligase, early Earth, autocatalytic sets, RNA world

## Abstract

The evolutionary origin of the genome remains elusive. Here, I hypothesize that its first iteration, the protogenome, was a multi-ribozyme RNA. It evolved, likely within liposomes (the protocells) forming in dry-wet cycling environments, through the random fusion of ribozymes by a ligase and was amplified by a polymerase. The protogenome thereby linked, in one molecule, the information required to seed the protometabolism (a combination of RNA-based autocatalytic sets) in newly forming protocells. If this combination of autocatalytic sets was evolutionarily advantageous, the protogenome would have amplified in a population of multiplying protocells. It likely was a quasispecies with redundant information, e.g., multiple copies of one ribozyme. As such, new functionalities could evolve, including a genetic code. Once one or more components of the protometabolism were templated by the protogenome (e.g., when a ribozyme was replaced by a protein enzyme), and/or addiction modules evolved, the protometabolism became dependent on the protogenome. Along with increasing fidelity of the RNA polymerase, the protogenome could grow, e.g., by incorporating additional ribozyme domains. Finally, the protogenome could have evolved into a DNA genome with increased stability and storage capacity. I will provide suggestions for experiments to test some aspects of this hypothesis, such as evaluating the ability of ribozyme RNA polymerases to generate random ligation products and testing the catalytic activity of linked ribozyme domains.

## 1. Introduction

The origin of life, i.e., the transition from abiotic molecules to replicating entities subject to Darwinian evolution, remains elusive. There are two main hypotheses that can be summarized as ‘RNA-first’ (the RNA world hypothesis), or, more broadly, ‘replicator-first’, and ‘metabolism-first’. The former (described in detail below) suggests that the ancestor of the genome arose spontaneously as a self-replicating oligo- or polymer of RNA, and that the metabolism emerged as a consequence of the evolving RNA. The latter hypothesis posits that a metabolism, i.e., a set of chemical reactions and their respective catalysts, within a primordial cell (the protocell) existed and evolved in complexity without any form of genome. Compelling arguments for both scenarios have been made in recent decades theoretically as well as by mathematical modeling and experimentation. Both hypotheses can arrive at some entity with the basic characteristics of life, i.e., the ability to multiply and to undergo Darwinian evolution. However, today’s cells contain two components, a replicating genome and a metabolism. A convincing scenario explaining both the evolution of the metabolism and the genome has not been formulated to the best of my knowledge.

**Figure 1:**
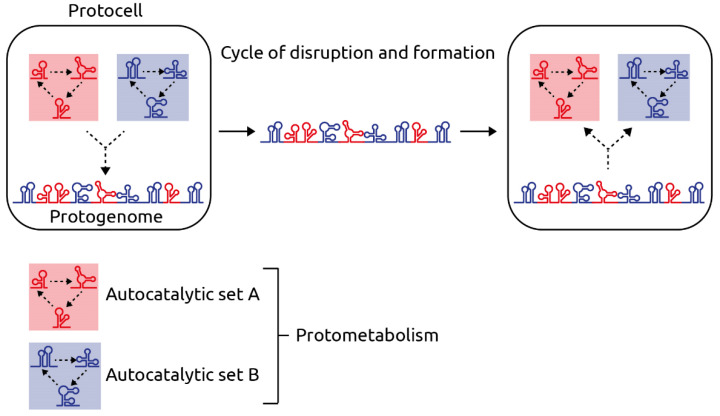
Schematic of the hypothetical protogenome and its function. In the left protocell (e.g., a liposome formed in a dry–wet cycle [6]), autocatalytic sets, composed of ribozymes, comprise a protometabolism. In this example, the protometabolism consists of two autocatalytic sets, A and B (red and blue, respectively). The protogenome, arising from the random ligation mediated by an RNA ligase ribozyme, contains, in one molecule, ribozyme domains of both autocatalytic sets. During a cycle of liposome disruption (‘dry’) and formation (‘wet’) the protogenome enters newly formed liposomes (right). There, it can seed both autocatalytic sets as its ribozyme domains are catalytically active. This process of protometabolism inheritance is more efficient than the stochastic co-encapsulation of individual ribozymes from autocatalytic sets A and B.

In the hypothesis presented here, I suggest that a protometabolism of catalytic RNAs (ribozymes) evolved first and gave rise to the first iteration of the genome, the protogenome. I will start by summarizing three key concepts underlying my hypothesis: the RNA world hypothesis [1], the concept of autocatalytic sets (i.e., a collection of molecules, each of which can be created through catalysis by other molecules within the set; see Figure 1) originally proposed by Kauffman [2,3,4] and dry–wet cycles as likely origin and early evolution of RNA polymers and liposome-based protocells (the compartments containing the autocatalytic sets and, subsequently, the protogenome), proposed by Henning and coworkers and Damer and Deamer, respectively [5,6]. I will then lay out my hypothesis on genome evolution, whose key aspect, the emergence of the protogenome from RNA-based autocatalytic sets, is summarized in Figure 1. I will conclude with ideas on how some of the aspects of this hypothesis might be tested experimentally.

### 1.1. The RNA World

The RNA world hypothesis suggests that there was a stage during Earth’s evolutionary history in which self-replicating RNA molecules proliferated [1]. From these initial RNA replicators, proteins, DNA, and ultimately all life on Earth evolved. The basis of this hypothesis is the observation that RNA is the only biopolymer known to be capable of both storing genetic information (as, for example, in RNA viruses) and of being catalytically active in the form of ribozymes. One can thus imagine a self-replicating RNA molecule that uses itself as a template. The first prerequisite for the emergence of such a replicator is the presence of ribonucleotides as building blocks in the ocean of early Earth.

Life on Earth likely evolved about four billion years ago [7]. The reducing atmosphere of early Earth lacked free oxygen and the primordial ocean presumably contained various organic compounds present in dilute concentrations in the micromolar range [8]. Simple organic molecules like hydrogen cyanide (HCN) or formaldehyde (CH_2_O) were likely formed from precursors such as water (H_2_O), methane (CH_4_), ammonia (NH_3_), and hydrogen (H_2_). The subsequent chemical reactions that yielded more complex organic molecules such as nucleobases, amino acids and lipid precursors must have required regions that allowed for a concentration of the reactants, creating the ‘warm little pond’ envisioned by Charles Darwin. Such regions may have been hydrothermal fields undergoing wet–dry cycles as proposed by Damer and Deamer (described below) [6] or hydrothermal vents with strong temperature gradients [9]. The abiotic synthesis of complex organic molecules has been achieved experimentally by simulating the likely conditions on early Earth, starting famously with the Miller–Urey experiment published in 1953 [10]. This experimental setup achieved the abiotic synthesis of various amino acids from a reducing atmosphere composed of CH_4_, NH_3_, H_2_ and water vapor, when energy was provided by electric discharge to simulate lightnings. Even though early Earth’s atmosphere, according to more recent data, might have looked different to the one simulated in the Miller–Urey experiment [11], these experiments provided first proof that complex organic molecules can arise abiotically. Following this, other groups have achieved the chemical synthesis of additional amino acids, as well as nucleotides and lipid precursors [12,13,14,15] (see [16] for a review). The abiotic chemical synthesis of ribonucleotides from simple precursors under prebiotically plausible conditions in 2009 provided further credence to the existence of the RNA world [14,17]. An additional source of complex organic molecules may have been meteorites; for example, a large array of organic compounds including nucleobases and amino acids have been identified in the Murchison meteorite [18].

The next step in the RNA world scenario is the formation of random RNA oligo- or polymers. Oligomers can form in solution [13,19] and on mineral surfaces [20]; polymers could have emerged in ponds undergoing dry–wet cycles [5], which is also a likely site for the other component required for life to emerge, evolving liposomes (see below). Chemically, initial RNA oligomerization may have been achieved by activated ribonucleotides [21,22] or activation agents [23,24]. Some of the random oligo- or polymers of RNAs could have been catalytically active and become part of autocatalytic sets, possibly together with other organic species such as peptides. It is plausible that the initial prebiotic ribozymes formed in such an environment of repeated dry–wet cycles; however, the exact mechanisms of initial oligonucleotide formation remain elusive. It is assumed here that oligonucleotides have formed abiotically.

The naturally occurring ribozymes in today’s world appear to have limited functionalities, mainly catalyzing peptide bond formation in ribosomes, as well as cleavage and ligation of phosphodiester bonds of RNAs [25,26,27,28]. In vitro evolution experiments, however, have generated ribozymes with various catalytic activities [29]. The first such identified ribozymes were DNA and RNA ligases [30,31]. One of the RNA ligases has been successfully evolved to an RNA-dependent RNA polymerase (RdRp), which, however, was only able to polymerize a maximum of 14 successive nucleotides [32]. A later iteration could incorporate up to 20 nucleotides [33]. More processive RdRps able to copy up to about 200 nucleotide RNAs have been reported subsequently [34,35,36,37,38]. In addition, a reverse transcriptase ribozyme (RNA-dependent DNA polymerase) has been identified by in vitro evolution, generating DNA products of up to 32 deoxynucleotides [39]. Other in vitro evolved ribozymes catalyze nucleotide synthesis [40], aminoacyl transfer [41], the hydrolysis of carboxyesters [42] and thiophosphates [43], as well as redox reactions [44,45].

Given this rich catalytic repertoire of ribozymes, the RNA world scenario seems plausible and even likely to have existed in the form of ribozyme-catalyzed metabolisms [46]. However, the evolution of the first replicator remains an unsolved problem, as no self-replicating RNA has been identified to date [47]. The closest to an experimentally verified self-replicating RNA system has been a set of two RNA ligase ribozymes that catalyze each other’s formation (a simple autocatalytic set), which however requires complex preformed RNA building blocks [48]. Moreover, if the first replicator was a self-replicating RdRp ribozyme, its spontaneous emergence is highly unlikely given that it would consist of about 200 nucleotides [35,49]. In addition, a spontaneously emerging RdRp is likely to be error-prone and therefore unlikely to enable stable self-replication [35,46]. Thus, the largest gap in the RNA world hypothesis remains the emergence of the initial self-replicating RNA.

### 1.2. Autocatalytic Sets—Evolution of Metabolism without a Genome

The concept of autocatalytic sets (also referred to as collectively autocatalytic sets) provides an explanation on how a metabolism can emerge and evolve without the need of a dedicated information storage device such as the genome. The idea was pioneered by Stuart Kauffman [2,3]. In an autocatalytic set, a set of catalysts (i.e., molecules catalyzing a chemical reaction) are arranged such that the formation of each catalyst is achieved by other catalysts in the set, food sources (building blocks) provided. Thus, the whole set is autocatalytic, as opposed to a single molecule as stated by the RNA world hypothesis. The simplest autocatalytic set is one of two molecules that mutually catalyze the formation of one another. Experimentally, such a system was created by Gerald Joyce and coworkers with two RNA ligase ribozymes catalyzing each other’s formation [48,50]. Earlier, von Kiedrowski and coworkers generated cross-catalytic sets of short nucleotide sequences [51,52]. A more complex autocatalytic set composed of up to 16 RNA molecules that assemble into a self-replicating set has been achieved by Lehman and coworkers [53]. New ribozyme functionalities have been successfully generated by recombination [54] and an RNA-based autocatalytic set that couples anabolism and catabolism has been reported [55]. More recently, it was shown experimentally that a ribozyme can diversify and achieve multiple functions within an autocatalytic set [56] and that there is a trade-off between reproductive fitness and variation, providing insights into the evolutionary dynamics of autocatalytic sets [57]. Of note, such an autocatalytic set can be composed of other biomolecules as well. For example, a complex peptide-based autocatalytic set has been reported [58]. Autocatalytic sets also exist in nature and have been, for example, identified in the metabolism of *Escherichia coli* [59], providing evidence that autocatalytic sets evolved naturally.

Mathematical and computational studies on autocatalytic sets, formally described as reflexively autocatalytic and food-generated (RAF) sets (with food being a set of building blocks such as organic molecules on early Earth) have shown that autocatalytic sets have a high probability of emerging even with moderately active catalysts [4]. Moreover, randomly generated autocatalytic sets often contain subsets that are themselves autocatalytic sets, which can become dependent on each other, recombine, and thereby become more complex [60,61]. However, how exactly autocatalytic sets of organic polymers could have evolved and been selected for is still unclear. Vasas et al. (2012) discussed this problem and found that the prerequisite for evolvability are chemical reactions networks with multiple viable cores [62]. Although it remains difficult to imagine theoretical autocatalytic sets capable of evolution by natural selection [63], it is assumed here that RNA-based autocatalytic sets have this ability.

In summary, autocatalytic sets have likely spontaneously emerged from prebiotic, organic precursors present on early Earth, were self-sufficient, replicating, and capable of evolution. RNA oligo- or polymers, whose precursors were likely present on early Earth [14], are a likely catalytic component of the early metabolic networks. However, sustaining such systems in the ocean of early Earth would have been unlikely because of diffusion of the components, especially since the ocean was likely a very dilute ‘primordial soup’ of organic molecules with concentrations in the micromolar range [6,8]. For the autocatalytic sets to be sustainable and capable of evolution, they needed to be confined in a container [61]. The most likely container for early life to evolve were liposomes, as discussed in the following section.

### 1.3. Dividing Liposomes

Today’s cellular organisms are physically contained by a membrane mainly composed of a bilayer of phospholipids (bacteria and eukaryotes) or ether lipids (archaea). It is thus likely that the precursors of cells, the protocells, were also surrounded by a lipid bilayer, i.e., a liposomal container [64]. Artificially generated liposomes can grow and divide, for example, by incorporating externally provided micelles composed of fatty acids [65,66] or by the ability of internal production of phospholipids [67]. The latter finding suggests that if a liposomal protocell that has the capability of synthesizing phospholipids (by means of autocatalytic sets) would also be able to divide, i.e., produce offspring. The building blocks for lipid synthesis were likely available on early Earth from abiotic synthesis [15]—but how did liposomes first emerge spontaneously?

An answer to this question has been provided by Damer and Deamer [6]. In areas on early Earth subject to dry–wet cycles, multilamellar lipid layers (during the dry cycle) form liposomes (during the wet cycles). If there are organic molecules in the aqueous phase, they will be distributed into the liposomes, where autocatalytic sets might come into existence. Thus, protocells can form that are able to evolve and, if capable of lipid synthesis, divide during the wet cycles. During the dry cycles, the contents of all the liposomes are combined and re-distributed into the emerging liposomes that form during the wet cycles.

## 2. Evolution of the Genome from RNA-Based Autocatalytic Sets

Now we have all the components that are required to form a protocell with an RNA-based protometabolism, as laid out above, consisting of RNA oligo- or polymers that form an autocatalytic set.

1. The abiotic synthesis of precursors for RNA and lipid molecules in chemically plausible conditions on early Earth has been experimentally verified (see Section 1.1);
2. Autocatalytic sets have a high probability of forming spontaneously, as per the mathematical RAF theory, and RNA (ribozyme)-based autocatalytic sets have been experimentally generated (see Section 1.2);
3. Liposomes can form spontaneously in dry–wet cycles and can grow, divide, and evolve (see Section 1.3). Such dry–wet cycles also allow for the oligomerization of RNA molecules [5].

Altogether, the dry–wet cycles appear to be a plausible scenario for the first protocells to evolve. Yet, to resemble present-day cells, these protocells require a genome that contains or encodes the information for the protometabolism.

### 2.1. Protogenome Evolution within the Dry–Wet Cycle Scenario

First, let me point out why a ribozyme-based protometabolism is more likely to evolve increasing complexity than one composed solely of peptides. RNAs are more likely to evolve into more complex molecules (i.e., more specific and more catalytically active ribozymes) because they can be complementary to each other and are less chemically diverse than peptides. This allows for replication of RNA species (e.g., by a polymerase) and sequence-specific cleaving and joining. Thus, it appears more plausible that, initially, ribozymes evolved, while peptide-based catalysts likely remained relatively simple, restricted to short oligomers. Moreover, before the evolution of a genetic code, the sequence-specific assembly of long peptides would have been limited in length due to the chemical diversity of amino acids, as their joining would have required highly specific catalysts (that could, for example, differentiate between aspartic acid and glutamic acid; and the number of amino acids available on early Earth was likely bigger than the 20 proteinogenic amino acids of today’s cells). Of note, these additional amino acids were of limited relevance for the evolution of protein biosynthesis whose code likely became ‘frozen’ in its present form, as originally proposed by Francis Crick [68,69]—I will further elaborate on this topic in the discussion (Section 3). Because of their chemical diversity and non-complementarity, amino acids likely required a genetic code to be assembled into complex enzymes.

Of note, ribozymes can be relatively small. Thus, simple representatives have likely emerged from the random oligomerization of ribonucleotides in dry–wet cycles. For example, the smallest ribozyme identified to date consists of only five nucleotides with a three-nucleotide active center, able to catalyze aminoacylation of another RNA [70], and minimal ligase ribozymes with catalytic cores as small as 18 nucleotides have been identified by in vitro evolution [71]. It is conceivable that such small ribozymes may have formed by the random oligomerization of RNAs in dry–wet cycles.

An early protocell might have contained autocatalytic sets composed of such small ribozymes. As the autocatalytic sets evolved, more complex ribozymes might have emerged. A ribozyme-based protometabolism likely contained the ribozymes required for RNA assembly and copying, i.e., RNA ligases and polymerases. The polymerase might have evolved from a ligase, similar to in vitro evolution experiments that have successfully generated polymerases from ligases [32].

**Figure 2:**
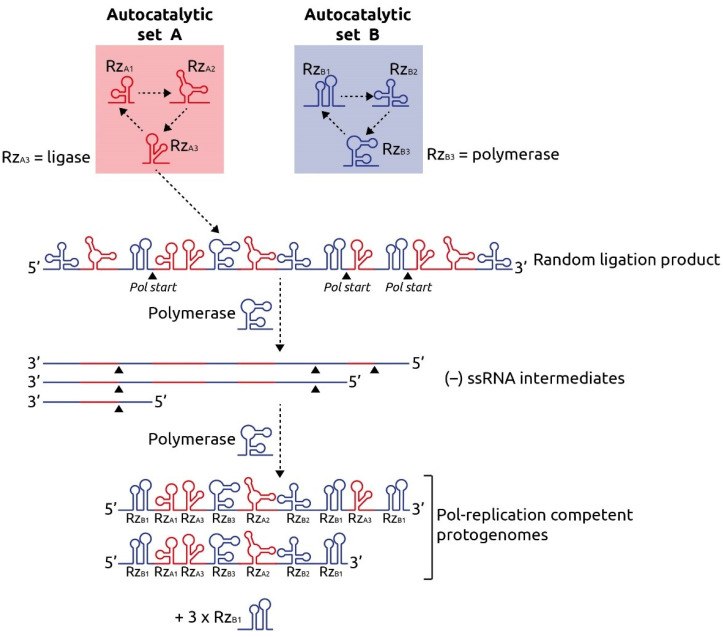
Emergence of the protogenome from RNA-based autocatalytic sets. The protometabolism in this example consists of two independent autocatalytic sets A and B, both comprising three ribozymes (Rz), Rz_A1_ through Rz_A3_ and Rz_B1_ through Rz_B3_, respectively. In this example, Rz_A3_ is an RNA ligase and Rz_B3_ an RNA polymerase. The RNA ligase produces random ligation products of the ribozymes such as the one depicted. Since the RNA polymerase also catalyzes the replication of Rz_B1_ from an autocatalytic set, there are start signals for the polymerase at any location where Rz_B1_ has been ligated into the RNA (indicated by upward arrowheads). The RNA polymerase generates the minus strand (−) ssRNA replication intermediates that also contain RNA polymerase start signals indicated by arrows. The plus strand copies are shown at the bottom; in this example these are two protogenome versions, both terminated by Rz_B1_ domains at the 5′ and 3′ ends, and three copies of Rz_B1_.

The first iteration of the genome precursor can be explained by means of the RNA ligase. The RNA ligase ribozymes are generally template-dependent and thereby restricted to an RNA substrate with a specific sequence. However, template-independent ligation might have occurred ‘accidentally’, leading to the random joining of two or more ribozymes. If the random joining of ribozymes occurred in such a way that, for example, it contained the ribozymes required to seed, in one molecule, two or more independent autocatalytic sets that collectively provided an evolutionary advantage over the individual autocatalytic sets, this multi-functional ribozyme protogenome could spread through a population of protocells. It would be reminiscent of a multi-domain protein, in which the individual domains provide a certain combined functionality. Moreover, such an RNA would be able to be replicated by means of an RNA polymerase if it contained two copies of the original ribozyme that was subject to copying by the polymerase (in the example herein designated as ‘Rz_B1_’, since Rz_B1_ contains start sites for polymerization (Figure 2)). The resulting protogenome, after polymerization, would have Rz_B1_ sequences at the 5′ and 3′ ends and the collection of randomly joined ribozymes in between. Importantly, it can combine in one RNA molecule ribozymes of different, potentially independent, autocatalytic sets (red and blue in Figure 2). Such a protogenome would have virus-like properties; the ends are identical in sequence (such as terminal repeats characteristic of, for instance, retroviruses). These ends are required as starting points for the RNA polymerase to generate the complementary RNA (‘minus’ strand) and the subsequent polymerization of the protogenome copy (‘plus’ strand) (see Figure 2 for details). Interestingly, the recently discovered retrozymes (a form of retrotransposons) have terminal repeats that contain hammerhead ribozyme domains, encompassing a region of non-coding RNA [72]. Thus, virus-like entities with similarities to the herein proposed protogenome still exist today. The protogenome would have represented an evolutionary disadvantage if it used up too many building blocks for its own replication. As such, versions of it that replicated relatively slowly (e.g., by mutations in the termini that reduced the affinity to the RNA polymerase) might have been selected for in the dry–wet cycle scenario. Various variants of the protogenome (different composition and length) were likely assembled spontaneously initially and were subject to evolutionary competition.

There are three prerequisites for the above scenario to be plausible. First, random ligation products of ribozymes must have a probability to occur. Second, the joined ribozymes must retain their catalytic activity when part of a longer RNA molecule. Third, there must have been an RNA polymerase with sufficient fidelity to copy such a long RNA without running into an error catastrophe.

Regarding the first point, template-independent ligation of single-stranded DNA and RNA molecules has been demonstrated to be catalyzed by T4 DNA ligase and an RNA ligase from the archaeon *Thermococcus kodakarensis*, respectively (both are protein enzymes) [73,74]. RNA ligase ribozymes, especially short ones, have been shown experimentally to be able to accept a broad range of substrates [75], yet, to the best of my knowledge, non-templated ribozyme-catalyzed RNA ligation has not been demonstrated experimentally to date. However, given the fact that ribozymes tend to be generally more promiscuous than protein enzymes [46], it is conceivable that ribozyme RNA ligases have existed that also performed random ligation as a side product.

For the second prerequisite there is evidence from naturally existing RNAs as well as from experiments that ribozymes can remain catalytically active when they are part of longer RNA molecules. For example, active hammerhead ribozyme domains are embedded in the larger circular ssRNA genomes of *Avsunviroidae* viroids [76], virus satellite circRNAs [77], and hepatitis delta virus [78], as well as in various retroelements such as short interspersed nuclear elements (SINEs) [79], Penelope-like elements [80], and retrozymes [81]. Moreover, various artificial RNAs with multiple active ribozyme domains have been successfully generated, including two-ribozyme constructs [82,83,84], RNAs with four [85,86], up to nine [87], and up to ten ribozyme domains [88]. Therefore, a protogenome combining in one molecule the necessary ribozyme activities to start a combination of autocatalytic sets appears to be structurally possible.

With respect to the third point, there are indeed length restrictions to the initial protogenome that are imposed by the fidelity of the RNA polymerase. The error threshold defines the maximum length of an RNA molecule beyond which deleterious mutations will accumulate, as proposed by Manfred Eigen [89]. For ribozymes, and therefore the protogenome, a relaxed error threshold that increases the error threshold by approximately seven- to eightfold has been suggested, as mutations affect structural properties less than sequence information (as different RNA sequences can adopt a similar fold) [90]. According to this concept, the so far most accurate in vitro-generated RNA polymerase ribozyme with an average fidelity of about 97% [36,37] would allow for the stable replication of an RNA of only up to 250 nucleotides. This would, however, already allow for a number of ribozymes to be assembled in the protogenome, especially if one considers that ribozymes can be as small as five nucleotides. However, if the protogenome were to incorporate complex ribozymes such as the polymerase itself (likely around 200 nucleotides in length), a polymerase with higher fidelity would be required for stable replication. In this regard, the naturally evolved RNA polymerase ribozyme may have had a higher fidelity than the in vitro-generated variants, and fidelity might have been selected for in the protometabolism to allow for copying longer, more complex ribozymes. Of note, Attwater and colleagues successfully increased the average fidelity of an RNA polymerase ribozyme from 91% to above 97% by selecting for ribozymes that incorporate fewer mismatched nucleotides [38]. An RNA polymerase with an evolutionarily plausible fidelity of 99%, for example, would increase the relaxed error threshold to about 750 nucleotides. A protogenome of this length would already be able to contain several complex ribozymes. The error-prone nature of an early RNA polymerase ribozyme may have been the basis for further genetic innovation by introducing random mutations altering the catalytic activity and specificity of ribozymes.

### 2.2. Protogenome Evolution Outside of the Dry–Wet Cycle Scenario

If a protocell ‘escaped’ the dry–wet cycle scenario and became a free-living entity in the primordial ocean, or in areas such as tidal pools with higher concentrations of the organic molecule food sources, the evolutionary dynamics would have changed. The ability for liposome division must have had already evolved, e.g., via a phospholipid synthesis pathway (see above). The protogenome would have been an advantage for transmitting the metabolic information to the daughter cell during protocell division.

Yet, for the protogenome to become stably inherited during protocell division, the protometabolism had to become dependent on the protogenome. One way might have been that a genetic code evolved in the protogenome that enabled the generation of a peptide catalyst which provided an evolutionary advantage to the protocell. The protogenome, as it would have existed as a quasispecies due to the error-prone replication by an RNA polymerase enzyme, and likely contained redundant information (e.g., several copies of the same ribozyme domain) could have provided a ‘playing field’ for such a genetic novelty to evolve. Of note, small peptides as short as four amino acids have been shown to be efficient catalysts [91,92,93]. Such a peptide could be encoded by only eight (with a doublet code that might have preceded the triplet code [94]) or twelve nucleotides (with a triplet code). Simple RNA or DNA template-directed peptide formation reactions have been shown experimentally [95,96,97]. Such encoded peptides do not necessarily need to be catalysts themselves; they might have instead bound to existing ribozymes of the protometabolism and enhanced or modified their catalytic activities. Indeed, it has been proposed that the first template-directed peptides that evolved were cofactors of ribozymes [98]. Such peptides were likely positively charged, with an affinity to the negatively charged RNA. Experimentally, it has been shown, for example, that the activities of an RNA polymerase ribozyme and a hammerhead ribozyme can be strongly enhanced by the presence of positively charged small peptides [99] or a viral RNA-binding protein with net positive charge [100], respectively.

Another dependency mechanism might have been the evolution of addiction modules or toxin–antitoxin systems, in which the antitoxin RNA or templated peptide might have been present in the protogenome and neutralized a toxin in the protometabolism. There is evidence that a certain class of such toxins, Small Alarmone Synthetases, was already present in the last universal common ancestor (LUCA) as they are found in both bacteria and archaea [101,102].

### 2.3. Further Evolution of the Protogenome—En Route to LUCA

In order to resemble present-day cellular genomes, the protogenome must have increased in size. As the RNAs of the protometabolism increased in complexity and size, entities resembling present-day transposable or virus-like elements might have emerged, acting cooperatively within the protocell, as proposed by Villarreal and Witzany [103]. They could have had the ability to integrate into the protogenome and thereby provide it with new genetic information. Ancient retroelements with ribozyme activity that are able to integrate into DNA genomes are the group II introns [104], and an artificial RNA composed of two ribozyme domains evolved in vitro had the ability to integrate into ssRNA [83]. Of note, the evolutionary ancestors of present-day viruses and transposable elements, many of which are capable of genomic integration, have likely existed before LUCA [105]. Another mechanism leading to increased protogenome size could have been duplication events of parts of the protogenome. The longer the protogenome became, the higher the fidelity of the RNA polymerase needed to be in order to increase the error threshold; thus, there might have been a co-evolution of increasing protogenome size and RNA polymerase fidelity. Enhanced fidelity might have been achieved, for example, by the evolution of RNA-binding peptides. In addition, it has been recently shown that the processivity of a ribozyme RdRp could be enhanced substantially by introducing a ‘clamping’ domain that prevents dissociation from the RNA template during polymerization [106]. This suggests that other domains, such as a proofreading exonuclease ribozyme, may also be added and could have evolved naturally, enhancing the fidelity of the RdRp. A protein RdRp eventually evolved, likely increasing the possible genome size to that of present-day RNA viruses, which is up to ~30,000 nucleotides. According to Aravind and colleagues, the common ancestor of present-day RdRPs was likely a simple ~40 amino-acid-long peptide with three beta-sheets and a loop with a conserved 5-amino acid motif essential for catalysis, which may have initially been a homodimeric cofactor of a ribozyme [107]. If encoded by a triplet code, this peptide would have required ~120 nucleotides, or ~80 nucleotides with a duplet code that may have preceded the triplet code evolutionarily [94]. The RNA polymerase enzyme likely evolved early, before the emergence of LUCA [108]. Even longer genomes required the evolution of DNA as the carrier of information, which is chemically more stable than RNA. This required the evolution of a reverse transcriptase, perhaps initially as a ribozyme [39] and later as a protein enzyme. The evolution of the DNA genome must have preceded LUCA; the largest RNA viruses, coronaviruses, encode less than twenty genes [109], whereas LUCA’s genome likely contained over 300 genes [110].

## 3. Discussion

It has been proposed previously that complex ribozymes such as the RNA polymerase might have emerged by the random ligation of smaller hairpin-loop structures, which was supported by in silico studies [111]. A similar scenario was proposed for the evolution of the ribosomal RNAs [112]. Here, I extended these ideas and argued that the ancient genome might have evolved in a similar way, likely in dry–wet scenarios on early Earth, which have been suggested as plausible sites for liposome formation, the evolution of metabolic protocells, and RNA polymers [5,6]. Initial RNA oligo- and polymers may have assembled in such a scenario, assembling into autocatalytic sets contained within liposome containers that form and dissolve repeatedly. Random ligation of ribozymes of RNA-based autocatalytic sets may have occurred by means of an RNA ligase ribozyme, as similar template-independent ligation products have been shown for protein ligases [73,74]. The resulting protogenome described herein linked, in one molecule, the information of several autocatalytic sets, such that it could efficiently seed an evolutionarily advantageous protometabolism to other protocells. It was subsequently replicated by an RNA polymerase, and presumably different versions were in evolutionary competition with each other initially. Transient compartmentalization, a characteristic of the dry–wet cycle scenario, may have helped in the early evolution of the protogenome by preventing extinction due to parasitic mutants [113]. Complexity of the most fit variants was then increased by random mutations, perhaps leading to the evolution of the genetic code, through integration of early RNA transposable elements, and duplication events. The fidelity of the RNA polymerase ribozyme increased via co-evolution with the protogenome as it became larger. Once the protometabolism became dependent on the protogenome by means of the evolution of a genetic code within the protogenome, or by addiction modules, the protogenome would have been stably inherited outside of the dry–wet cycle environment. Of note, a crucial step towards free-living protocells in general, and LUCA in particular, is the evolution of the genetic code and proteins, hallmark features of all known cellular life. Protein biogenesis requires the genetic code and both likely evolved in an RNA world scenario [114]. While the exact evolutionary origin of the genetic code remains elusive, one common theory suggests that the code (the association between codons and amino acids) was arbitrary initially and subsequently evolved by expansion [68,69,115,116,117]. Once a point was reached when no further expansion was possible, it was ‘frozen’, resulting in the present genetic code. Scenarios for the evolutionary origin of the genetic code have been formulated. For example, the ‘coding coenzyme handle hypothesis’ states that anticodon-like RNA adaptors charged with amino acids served as coenzymes for ribozymes in an RNA world, leading to the evolutionary selection of codon–anticodon pairs [118]. Fitting within this framework, it has been suggested that the initial codons may have been solely composed of two nucleobases (G and C) capable of encoding four amino acids including alanine, followed by an ‘alanine world’ with codons composed of G, C and A nucleobases and the corresponding amino acids all chemically related to alanine [114,119]. Finally, the full set of codons (composed of G, C, A and U) added the remaining proteinogenic amino acids. These scenarios bridge the evolutionary transition from an RNA world with non-coding protogenomes to the present-day cellular world in which the vast majority of catalysts are genome-encoded proteins.

The herein-described hypothesis is supported by computer simulation studies which have confirmed that compartments such as protocells can be favorable for linkage of smaller RNAs to longer strands to occur evolutionarily [120,121] and that in such a system linkage to a protogenome supports more efficient ribozyme evolution [122]. Moreover, simulations indicate that the protogenome needs to be required for protocell replication in order to be stably inherited [122] and that the establishment and maintenance of protogenomes (linked genes) is under positive selection in protocells harboring non-linked genes [123,124].

Some of the aspects of this hypothesis could be tested experimentally. For example, are any of the in vitro generated RNA ligase ribozymes [29] able to generate random ligation products similar to protein-based ligases [73,74]? This could be tested by providing the RNA ligase with a pool of random RNA oligomers, or longer ribozymes, followed by deep sequencing of the reaction. It could also be tested if the ribozymes of two different known RNA-based autocatalytic sets, when combined in one RNA molecule, retain their catalytic activities and can be used as a starting point for both autocatalytic sets. These relatively simple experiments could provide further credence for the plausibility of the herein suggested hypothesis on the evolution of the protogenome.
